# Differential Impact of Zearalenone on Hepatic Glucose and Lipid Metabolism in Healthy and Ketotic Dairy Cows: An In Vitro Study

**DOI:** 10.3390/ijms26146827

**Published:** 2025-07-16

**Authors:** Justyna Barć, Zygmunt Maciej Kowalski, Wojciech Jagusiak

**Affiliations:** 1 Department of Animal Nutrition and Fisheries, University of Agriculture in Krakow, Al. Mickiewicza 24/28, 30-059 Krakow, Poland; rzkowals@cyf-kr.edu.pl; 2 Department of Animal Genetics, Breeding and Ethology, University of Agriculture in Krakow, Al. Mickiewicza 24/28, 30-059 Krakow, Poland; wojciech.jagusiak@urk.edu.pl

**Keywords:** zearalenone (ZEN), ketosis, dairy cows, hepatocytes, gene expression, glucose metabolism, lipid metabolism, in vitro model, mycotoxin toxicity, metabolic status

## Abstract

Zearalenone (ZEN), a mycotoxin commonly present in maize-based feed, poses a health risk to dairy cows. While the hepatic effects of ZEN are increasingly studied, little is known about its impact on cows with altered energy metabolism. This study investigated the transcriptional response of liver cells isolated from healthy and ketotic cows to ZEN exposure using a novel in vitro model. Hepatocytes were obtained via biopsy from 12 cows, cultured under standardized conditions, and exposed to ZEN (0–100 µM) for 1, 3, and 6 h. Gene expression analysis focused on targets related to glucose and lipid metabolism. ZEN induced time- and dose-dependent changes in gene expression, with the most prominent effects observed after 1 h. Key metabolic genes were differentially regulated depending on the cow’s metabolic status. Notably, hepatocytes from healthy cows showed a stronger transcriptional response than those from ketotic cows, indicating reduced metabolic adaptability in energy-compromised animals. Significant interaction effects between ZEN dose and metabolic status were observed, especially for genes involved in glycolysis and fatty acid metabolism. This study presents a novel in vitro model and emphasizes the need to consider metabolic health when assessing the risks of mycotoxin exposure in dairy cattle.

## 1. Introduction

Mycotoxins represent a significant challenge in agriculture and livestock production worldwide. Among the various mycotoxins produced by *Fusarium* fungi, Zearalenone (ZEN) poses a considerable risk not only for monogastric animals but also for ruminants [1]. ZEN contamination occurs primarily in cereal grains, including those in maize silage, which is a common component of dairy cow diets [2].

ZEN can be produced under favorable conditions of temperature and humidity, and its occurrence in feeds can vary significantly between regions and seasons [3]. It has been reported that ZEN levels in maize silage can increase during storage, especially if the silage is not properly ensiled or stored under suboptimal conditions [4]. The presence of ZEN in dairy cow diets is particularly worrisome because cows consume large quantities of feed, potentially leading to chronic exposure even at low contamination levels [5].

Although ZEN is mostly known for its estrogenic activity in swine, it may also negatively affect the reproduction of dairy cows [3,6]. The pathophysiological impact of ZEN in dairy cows is not fully understood, especially regarding cows suffering from metabolic disorders such as ketosis. Ketosis remains a prevalent metabolic disease of dairy cows, characterized by elevated concentrations of ketone bodies (acetoacetate, β-hydroxybutyrate, acetone) in the blood, urine, and milk [7]. It is associated with a negative energy balance [8]. Clinical and subclinical ketosis is associated with reduced milk yield, impaired reproductive performance, and increased susceptibility to other diseases [9,10]. Despite substantial research on ketosis, the potential interactions between ketosis and mycotoxin exposure, including ZEN, have not been comprehensively addressed. This gap in knowledge is critical because cows with metabolic disturbances may have altered detoxification mechanisms, such as impaired hepatic biotransformation capacity (e.g., reduced activity of phase I and phase II detoxification enzymes) or diminished antioxidant defenses, which can lead to a heightened susceptibility to toxins [11].

Previous studies have shown that ZEN can disrupt endocrine functions [12] and liver metabolism [11], yet the specific effects of ZEN on the hepatic cells of healthy versus ketotic cows remain largely unexplored. Since the liver plays a central role in both glucose and lipid metabolism, it is crucial to investigate whether ZEN exposure could exacerbate metabolic dysregulation in ketotic cows. Previous studies have shown that ZEN and its metabolites, such as α-zearalenol, can interfere with endocrine and metabolic functions, including glucose and lipid metabolism, in dairy cows [13,14]. These disruptions could potentially intensify the metabolic challenges already faced by ketotic cows. Understanding these interactions is essential for developing effective management strategies to mitigate the risks associated with mycotoxin exposure in metabolically vulnerable animals.

The present study aimed to evaluate the effects of ZEN on bovine liver cells isolated from biopsies obtained from healthy cows and cows with ketosis using a previously established cell culture model [15]. The study focused on assessing the expression of genes involved in glucose and lipid metabolism, as these pathways are known to be significantly affected during the development of ketosis in dairy cows. The selected genes related to glucose metabolism (*ENO1*, *PDHB*, *PGAM1*, *PGK1*, *TPI1*) encode key enzymes involved in glycolysis and pyruvate metabolism, which are essential for maintaining cellular energy balance. Alterations in their expression may reflect impaired glucose utilization, which is a hallmark of negative energy balance and metabolic stress.

In parallel, genes associated with lipid metabolism (*ACOX1*, *ACAA1*, *ACACA*, *FADS2*, *FASN*, *HMGCR*, *SC4MOL*) were included due to their roles in fatty acid oxidation, synthesis, and cholesterol biosynthesis. Changes in the expression of these genes can indicate shifts in hepatic lipid processing, commonly observed in ketotic animals. The selection of these specific genes was based on existing transcriptomic studies that reported their dysregulation in the liver of cows during ketosis and under mycotoxin exposure [16].

## 2. Results

### 2.1. Gene Expression Related to Glucose Metabolism

**Figure 1:**
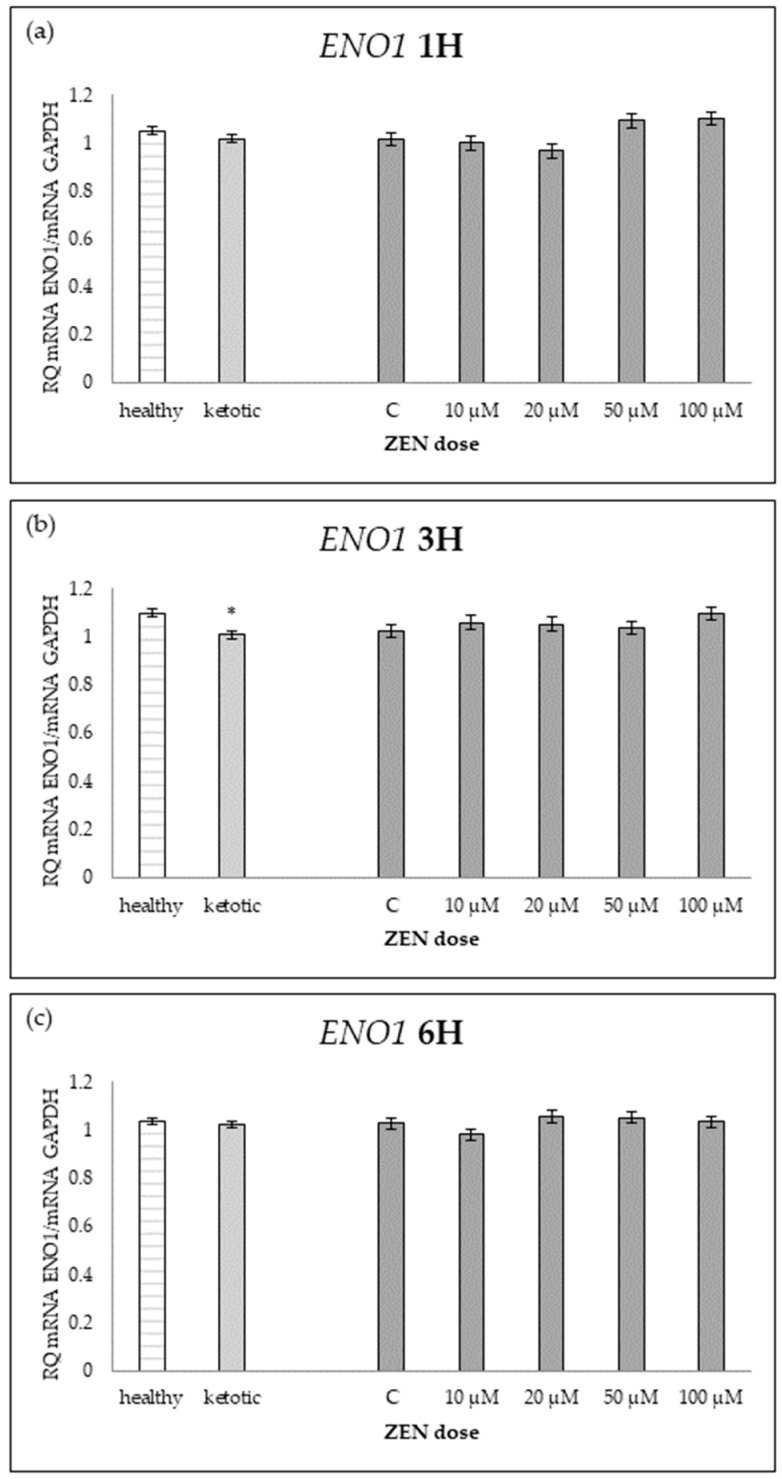
Relative mRNA expression of *ENO1* normalized to *GAPDH* in hepatocyte cultures derived from healthy and ketotic cows after exposure to Zearalenone (ZEN) for (**a**) 1 h, (**b**) 3 h, and (**c**) 6 h. Bars represent the mean relative quantity (RQ) of mRNA expression in control (C; 0 μM ZEN) and treatment groups (10, 20, 50, 100 μM). The expression in untreated control samples (C) was used as the calibrator for each group. Asterisk (*) indicates statistically significant differences between healthy and ketotic cows at the same time point (*p* < 0.05). Error bars represent standard error of the mean (SEM).

**Figure 2:**
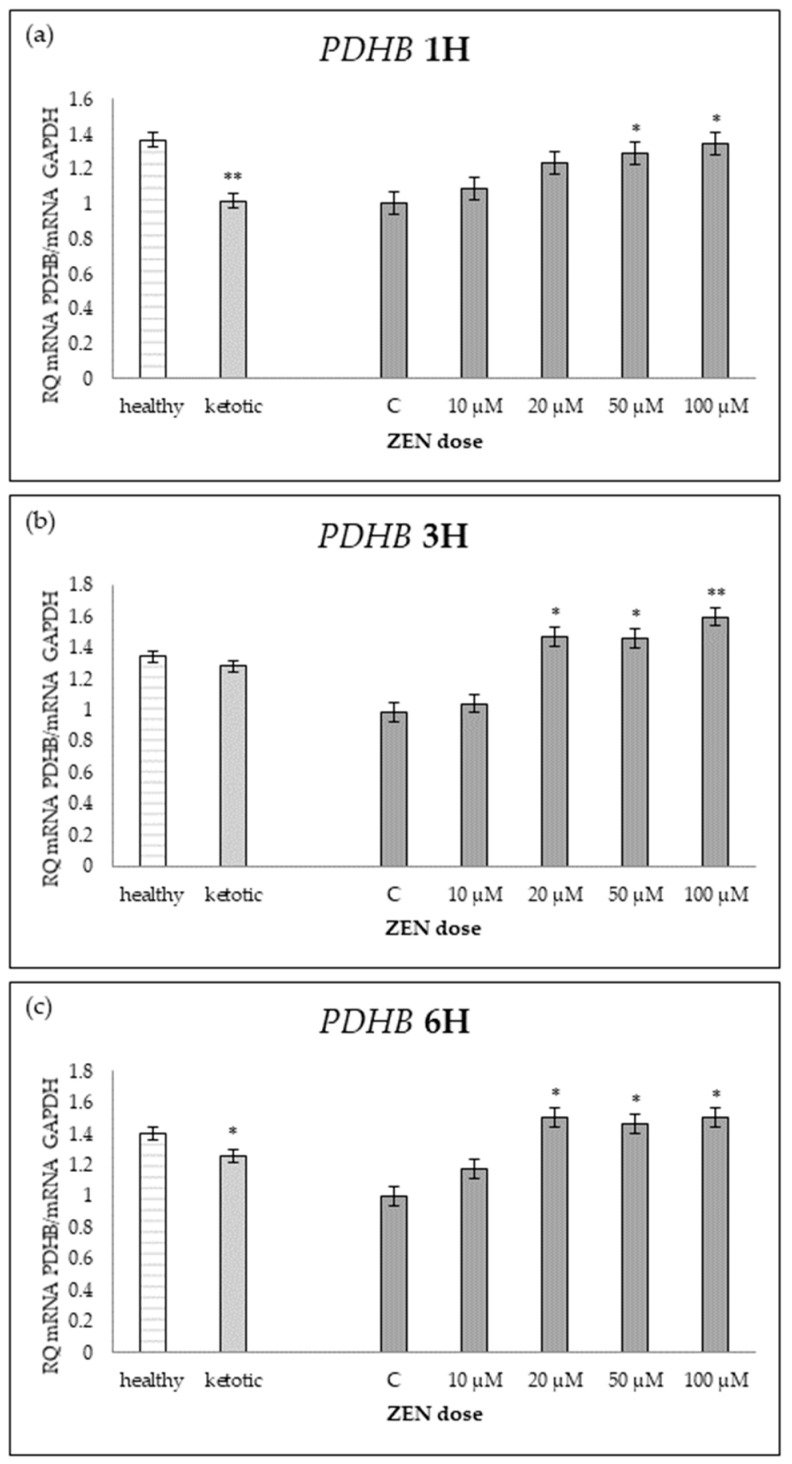
Relative mRNA expression of *PDHB* normalized to *GAPDH* in hepatocyte cultures derived from healthy and ketotic cows after exposure to Zearalenone (ZEN) for (**a**) 1 h, (**b**) 3 h, and (**c**) 6 h. Bars represent the mean relative quantity (RQ) of mRNA expression in control (C; 0 μM ZEN) and treatment groups (10, 20, 50, 100 μM). The expression in untreated control samples (C) was used as the calibrator for each group. Asterisks indicate statistically significant differences compared to the control within the same group: * *p* < 0.05, ** *p* < 0.001. Error bars represent the standard error of the mean (SEM).

**Figure 3:**
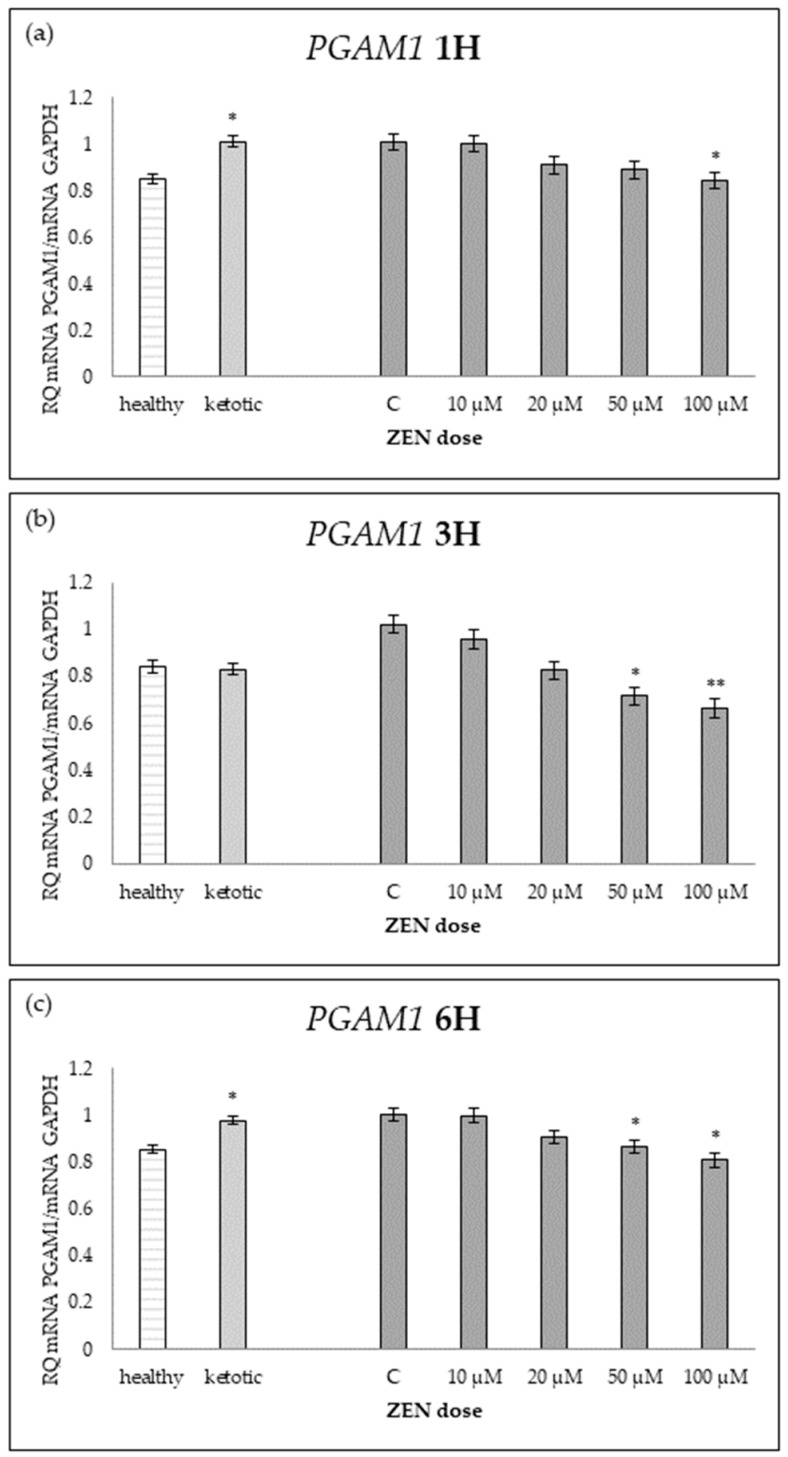
Relative mRNA expression of *PGAM1* normalized to *GAPDH* in hepatocyte cultures derived from healthy and ketotic cows after exposure to Zearalenone (ZEN) for (**a**) 1 h, (**b**) 3 h, and (**c**) 6 h. Bars represent the mean relative quantity (RQ) of mRNA expression in control (C; 0 μM ZEN) and treatment groups (10, 20, 50, 100 μM). The expression in untreated control samples (C) was used as the calibrator for each group. Asterisks indicate statistically significant differences compared to the control within the same group: * *p* < 0.05, ** *p* < 0.001. Error bars represent the standard error of the mean (SEM).

**Figure 4:**
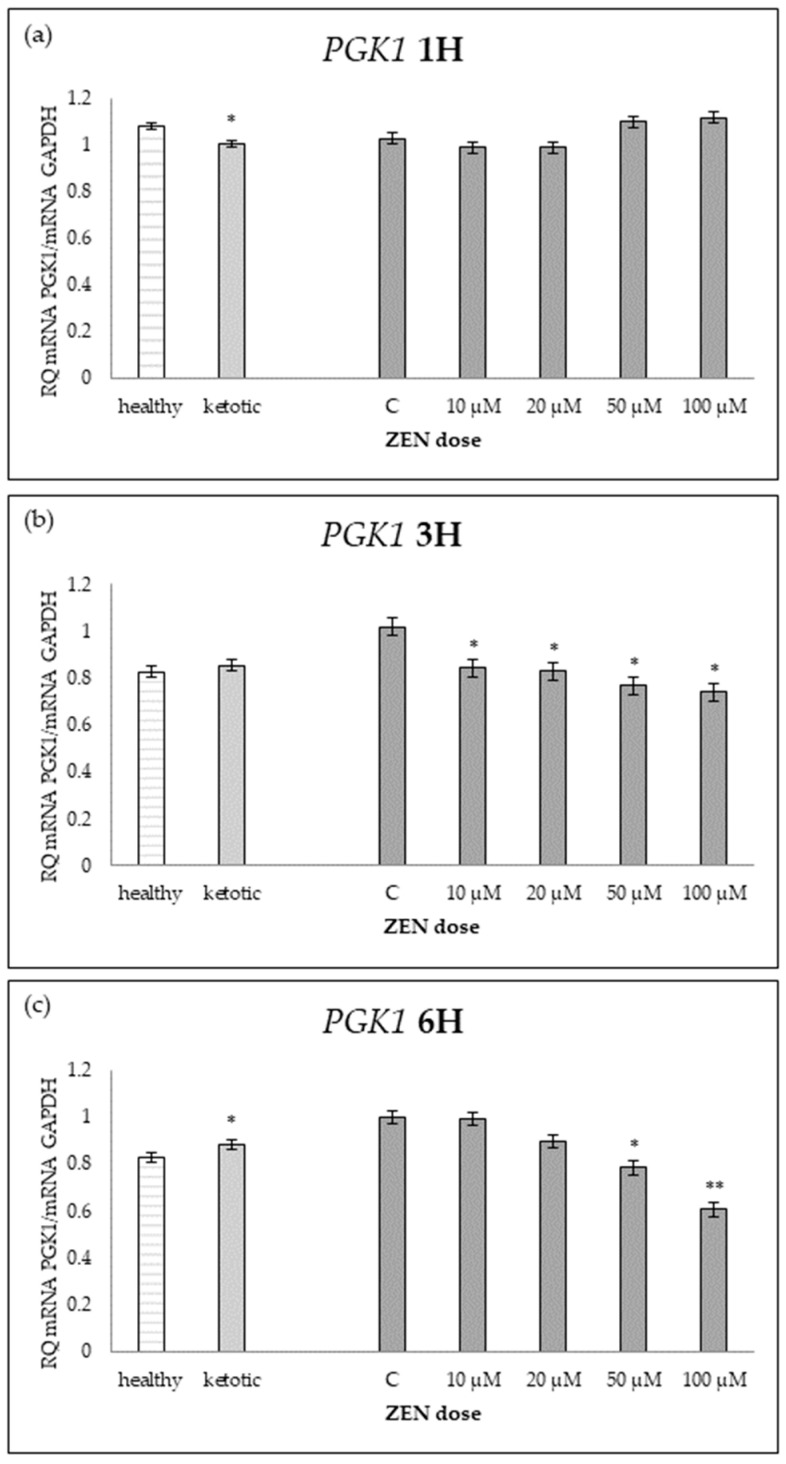
Relative mRNA expression of *PGK1* normalized to *GAPDH* in hepatocyte cultures derived from healthy and ketotic cows after exposure to Zearalenone (ZEN) for (**a**) 1 h, (**b**) 3 h, and (**c**) 6 h. Bars represent the mean relative quantity (RQ) of mRNA expression in control (C; 0 μM ZEN) and treatment groups (10, 20, 50, 100 μM). The expression in untreated control samples (C) was used as the calibrator for each group. Asterisks indicate statistically significant differences compared to the control within the same group: * *p* < 0.05, ** *p* < 0.001. Error bars represent the standard error of the mean (SEM).

**Figure 5:**
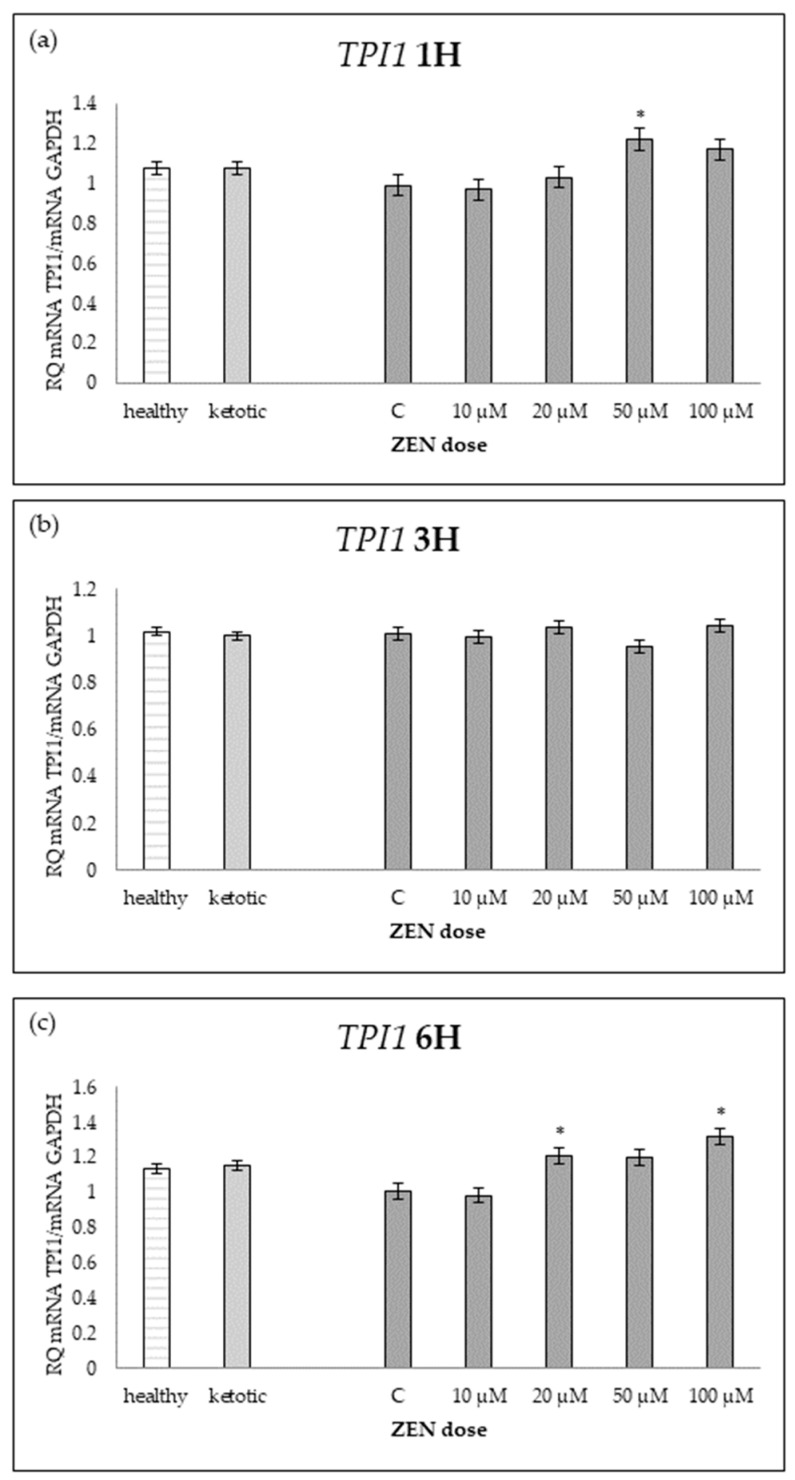
Relative mRNA expression of *TPI1* normalized to *GAPDH* in hepatocyte cultures derived from healthy and ketotic cows after exposure to zearalenone (ZEN) for (**a**) 1 h, (**b**) 3 h, and (**c**) 6 h. Bars represent the mean relative quantity (RQ) of mRNA expression in control (C; 0 μM ZEN) and treatment groups (10, 20, 50, 100 μM). The expression in untreated control samples (C) was used as the calibrator for each group. Asterisk (*) indicates statistically significant differences between healthy and ketotic cows at the same time point (*p* < 0.05). Error bars represent the standard error of the mean (SEM).

The expression of genes associated with glucose metabolism was influenced by both the metabolic status (MS) of the cows and exposure to increasing doses of ZEN (Table 1, Table 2, Table 3, Table 4 and Table 5, Figure 1, Figure 2, Figure 3, Figure 4 and Figure 5).

Cows in ketosis showed higher expression of *ENO1* and *PGAM1*, and lower expression of *PDHB*, compared to healthy cows (Table 1, Table 2 and Table 3; Figure 1, Figure 2 and Figure 3). In contrast, the expression of *PGK1* and *TPI1* was not significantly different between MS groups (Table 4 and Table 5; Figure 4 and Figure 5).

ZEN exposure affected the expression of all genes except *ENO1* (Table 1; Figure 1). ZEN induced a dose-dependent upregulation of *PDHB* and *TPI1*, while it decreased the expression of *PGAM1* and *PGK1*, particularly in healthy cows (Table 2, Table 3, Table 4 and Table 5; Figure 2, Figure 3, Figure 4 and Figure 5). These effects were more evident at higher ZEN doses and longer exposure durations.

For *PDHB* and *PGK1* the greatest changes were observed already after 1H of exposure to ZEN (Table 2 and Table 4), while for *PGAM1* and *TPI1* expression differences remained evident across all time points (Table 3 and Table 5; Figure 3 and Figure 5).

The interaction between MS and ZEN dose affected the expression of *PDHB* and *PGAM1* (Table 2 and Table 3), and the response to ZEN differed depending on the MS. In healthy cows, *PDHB* was strongly upregulated, and *PGAM1* was markedly downregulated, while ketotic cows showed more stable expression levels in response to ZEN. No interaction was observed for *ENO1*, *PGK1*, or *TPI1* (Table 1, Table 4 and Table 5).

### 2.2. Gene Expression Related to Lipid Metabolism

**Figure 6:**
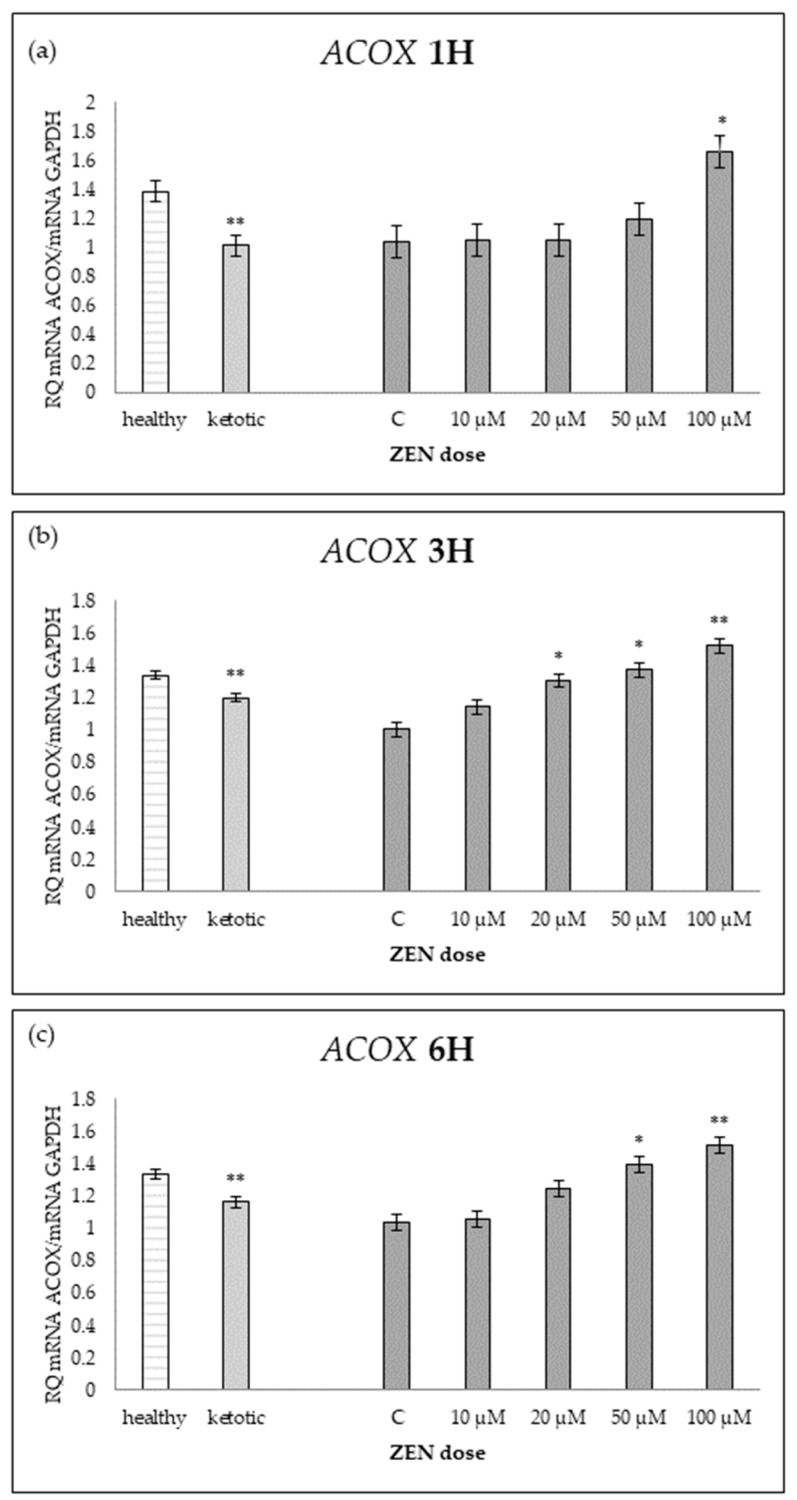
Relative mRNA expression of *ACOX1* normalized to *GAPDH* in hepatocyte cultures derived from healthy and ketotic cows after exposure to zearalenone (ZEN) for (**a**) 1 h, (**b**) 3 h, and (**c**) 6 h. Bars represent the mean relative quantity (RQ) of mRNA expression in control (C; 0 μM ZEN) and treatment groups (10, 20, 50, 100 μM). The expression in untreated control samples (C) was used as the calibrator for each group. Asterisks indicate statistically significant differences compared to the control within the same group: * *p* < 0.05, ** *p* < 0.001. Error bars represent the standard error of the mean (SEM).

**Figure 7:**
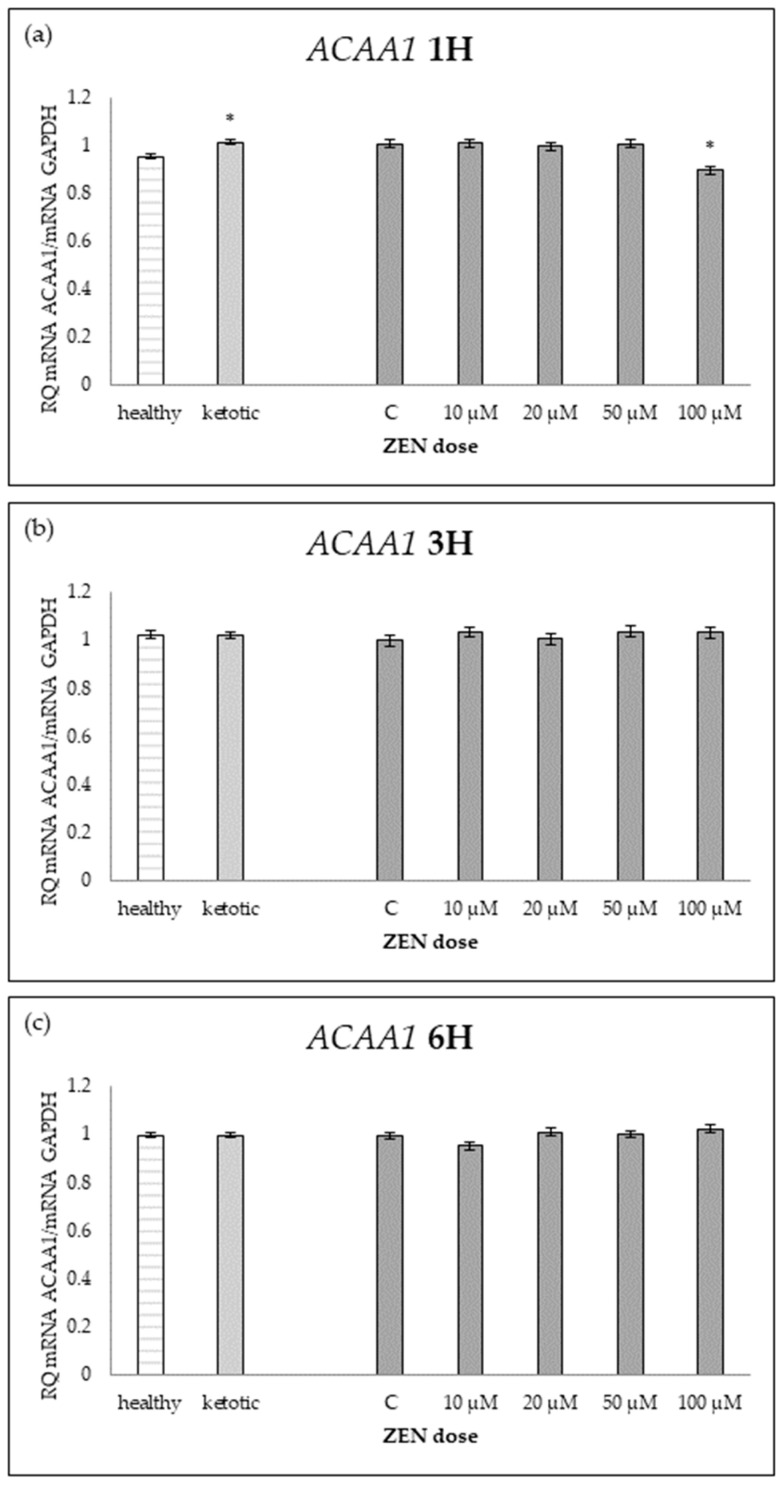
Relative mRNA expression of *ACAA1* normalized to *GAPDH* in hepatocyte cultures derived from healthy and ketotic cows after exposure to zearalenone (ZEN) for (**a**) 1 h, (**b**) 3 h, and (**c**) 6 h. Bars represent the mean relative quantity (RQ) of mRNA expression in control (C; 0 μM ZEN) and treatment groups (10, 20, 50, 100 μM). The expression in untreated control samples (C) was used as the calibrator for each group. Asterisk (*) indicates statistically significant differences between healthy and ketotic cows at the same time point (*p* < 0.05). Error bars represent the standard error of the mean (SEM).

**Figure 8:**
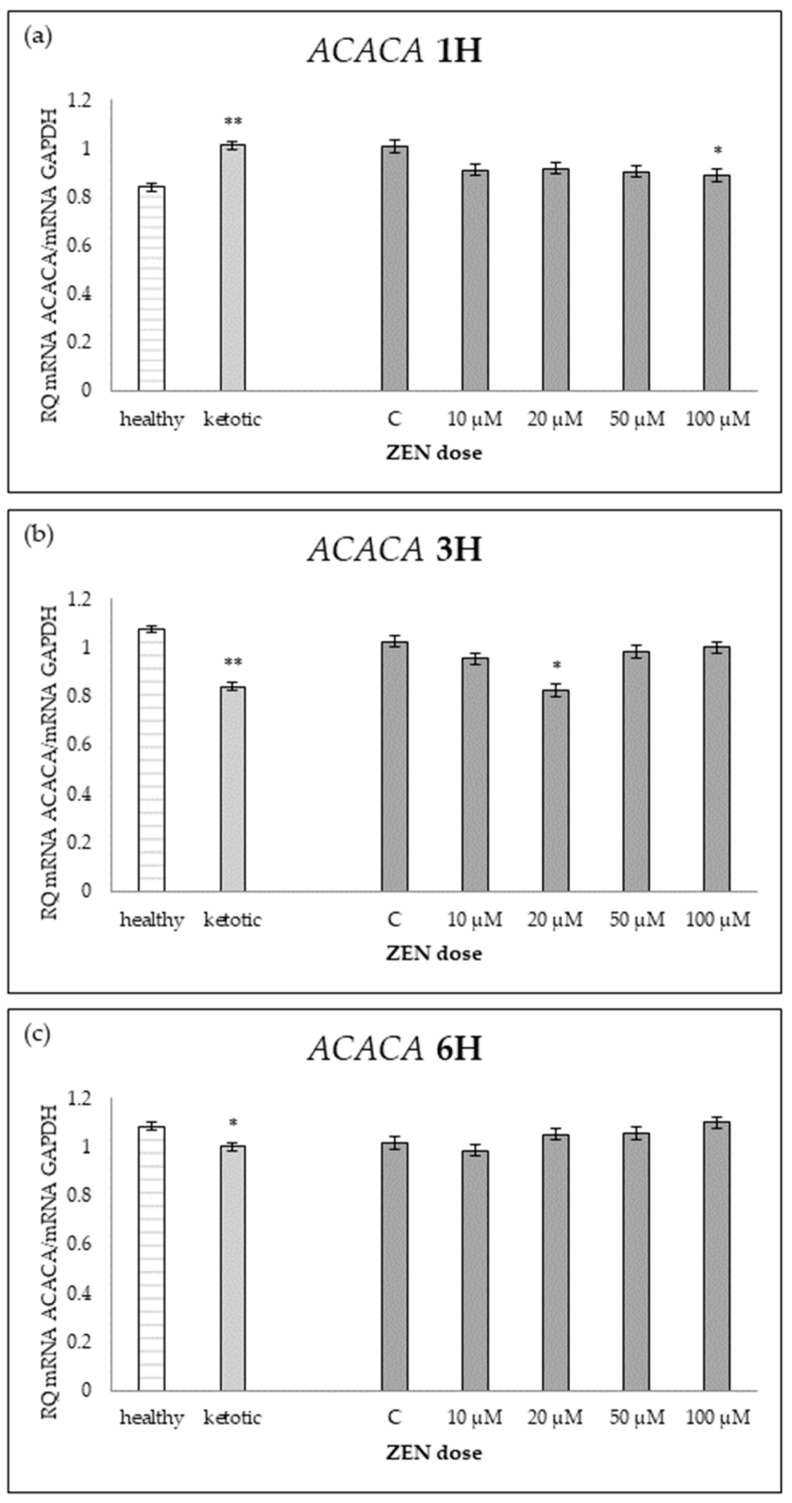
Relative mRNA expression of *ACACA* normalized to *GAPDH* in hepatocyte cultures derived from healthy and ketotic cows after exposure to zearalenone (ZEN) for (**a**) 1 h, (**b**) 3 h, and (**c**) 6 h. Bars represent the mean relative quantity (RQ) of mRNA expression in control (C; 0 μM ZEN) and treatment groups (10, 20, 50, 100 μM). The expression in untreated control samples (C) was used as the calibrator for each group. Asterisks indicate statistically significant differences compared to the control within the same group: * *p* < 0.05, ** *p* < 0.001. Error bars represent the standard error of the mean (SEM).

**Figure 9:**
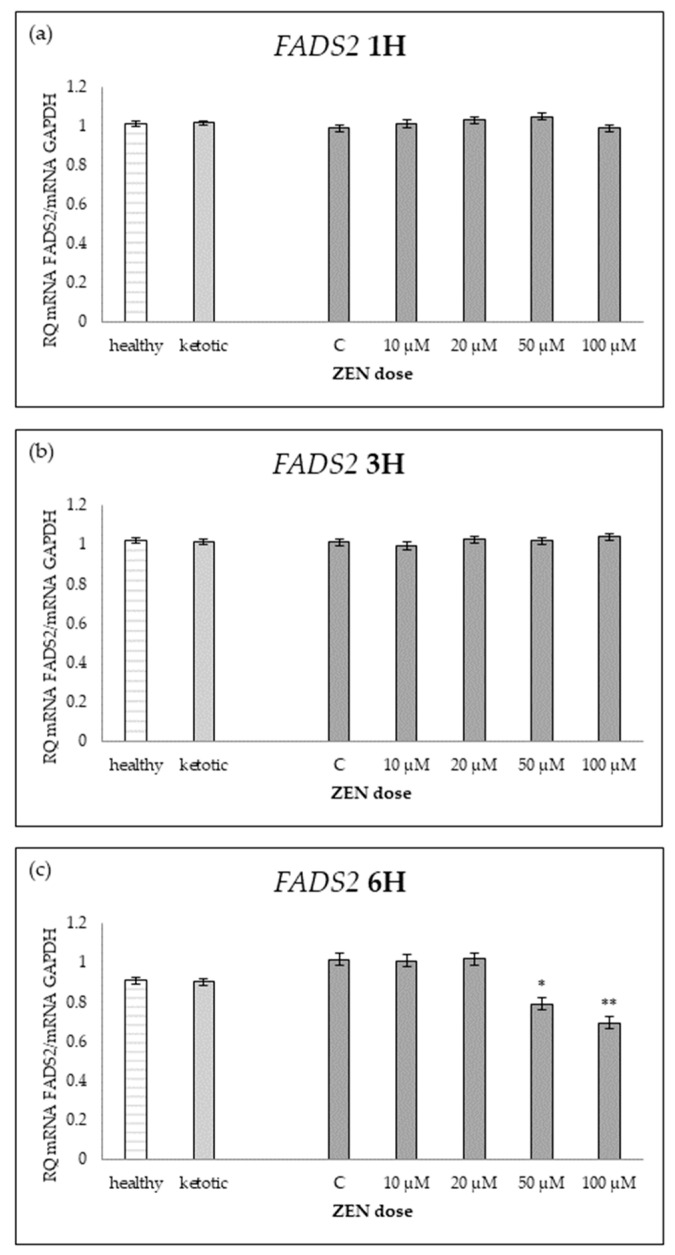
Relative mRNA expression of *FADS2* normalized to *GAPDH* in hepatocyte cultures derived from healthy and ketotic cows after exposure to zearalenone (ZEN) for (**a**) 1 h, (**b**) 3 h, and (**c**) 6 h. Bars represent the mean relative quantity (RQ) of mRNA expression in control (C; 0 μM ZEN) and treatment groups (10, 20, 50, 100 μM). The expression in untreated control samples (C) was used as the calibrator for each group. Asterisks indicate statistically significant differences compared to the control within the same group: * *p* < 0.05, ** *p* < 0.001. Error bars represent the standard error of the mean (SEM).

**Figure 10:**
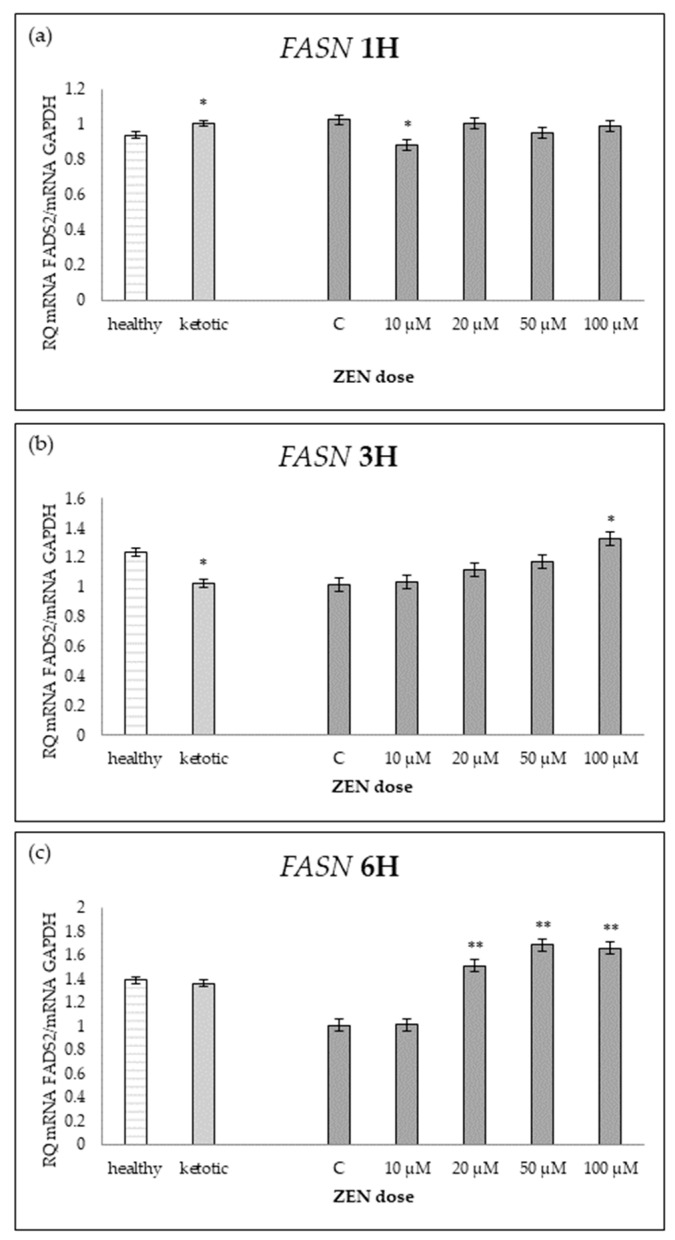
Relative mRNA expression of *FASN* normalized to *GAPDH* in hepatocyte cultures derived from healthy and ketotic cows after exposure to zearalenone (ZEN) for (**a**) 1 h, (**b**) 3 h, and (**c**) 6 h. Bars represent the mean relative quantity (RQ) of mRNA expression in control (C; 0 μM ZEN) and treatment groups (10, 20, 50, 100 μM). The expression in untreated control samples (C) was used as the calibrator for each group. Asterisks indicate statistically significant differences compared to the control within the same group: * *p* < 0.05, ** *p* < 0.001. Error bars represent the standard error of the mean (SEM).

**Figure 11:**
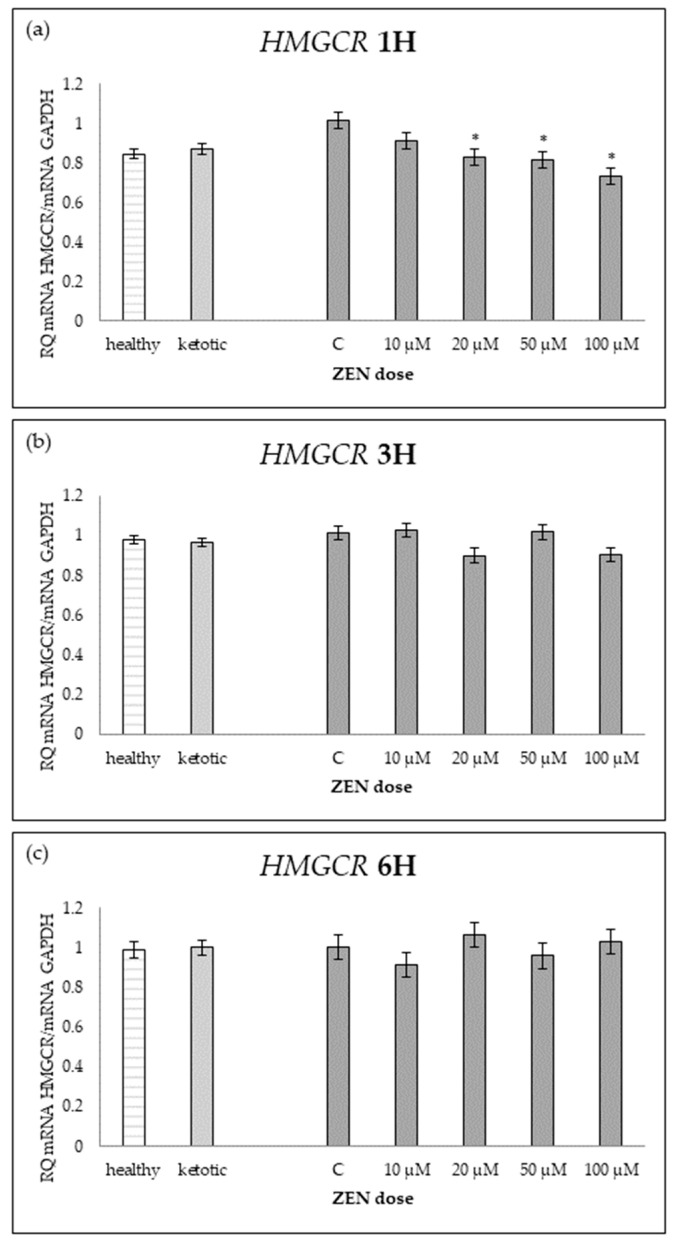
Relative mRNA expression of *HMGCR* normalized to *GAPDH* in hepatocyte cultures derived from healthy and ketotic cows after exposure to zearalenone (ZEN) for (**a**) 1 h, (**b**) 3 h, and (**c**) 6 h. Bars represent the mean relative quantity (RQ) of mRNA expression in control (C; 0 μM ZEN) and treatment groups (10, 20, 50, 100 μM). The expression in untreated control samples (C) was used as the calibrator for each group. Asterisk (*) indicates statistically significant differences between healthy and ketotic cows at the same time point (*p* < 0.05). Error bars represent the standard error of the mean (SEM).

**Figure 12:**
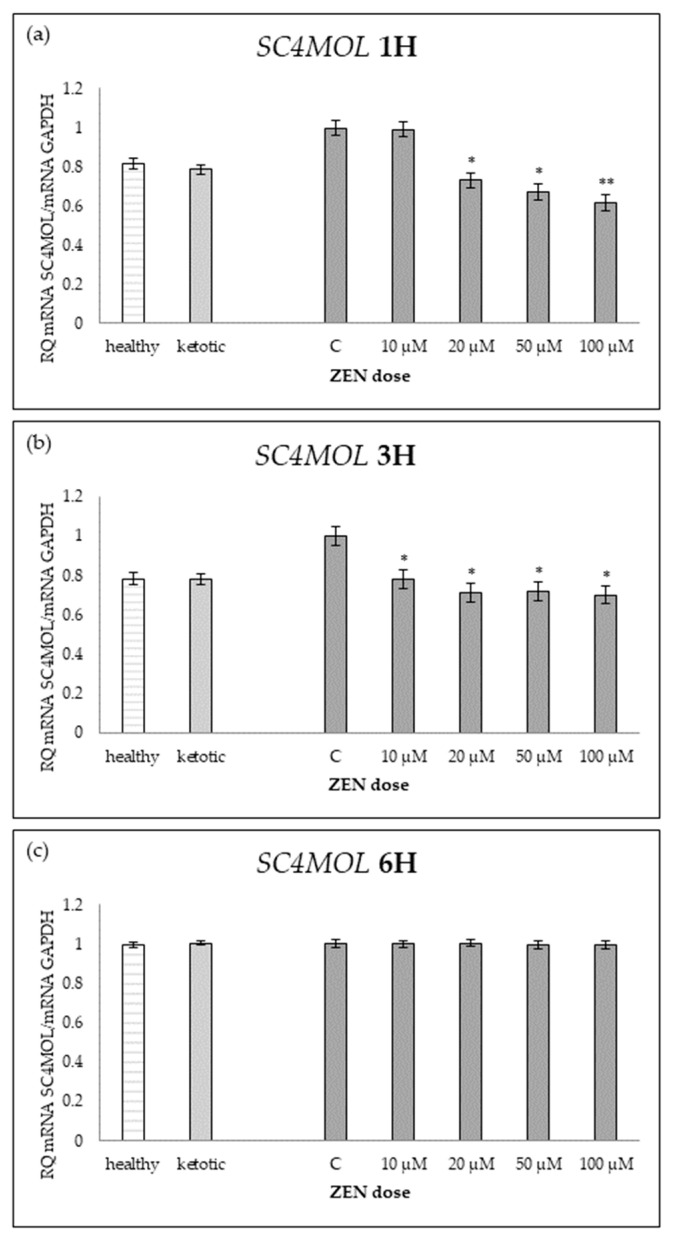
Relative mRNA expression of *HMGCR* normalized to *GAPDH* in hepatocyte cultures derived from healthy and ketotic cows after exposure to zearalenone (ZEN) for (**a**) 1 h, (**b**) 3 h, and (**c**) 6 h. Bars represent the mean relative quantity (RQ) of mRNA expression in control (C; 0 μM ZEN) and treatment groups (10, 20, 50, 100 μM). The expression in untreated control samples (C) was used as the calibrator for each group. Asterisks indicate statistically significant differences compared to the control within the same group: * *p* < 0.05, ** *p* < 0.001. Error bars represent the standard error of the mean (SEM).

The expression of genes associated with lipid metabolism was influenced by both MS and ZEN. Among the analyzed genes, *ACOX1*, *ACAA1*, *ACACA*, *FADS2*, *FASN*, *HMGCR*, and *SC4MOL* showed distinct expression patterns depending on the experimental factors (Table 6, Table 7, Table 8, Table 9, Table 10, Table 11 and Table 12, Figure 6, Figure 7, Figure 8, Figure 9, Figure 10, Figure 11 and Figure 12). Healthy cows showed higher expression of *ACOX1* and *FASN*, and lower expression of *ACACA*, compared to ketotic cows (Table 6, Table 8 and Table 10; Figure 6, Figure 8 and Figure 10). In contrast, the expression of *ACAA1*, *FADS2*, *HMGCR*, and *SC4MOL* was not different between groups (Table 7, Table 9, Table 11 and Table 12; Figure 7, Figure 9, Figure 11 and Figure 12).

ZEN induced a dose-dependent increase in *ACOX1* and *FASN* expression, while it decreased the expression of *SC4MOL* (Table 6, Table 10 and Table 12; Figure 6, Figure 10 and Figure 12). In healthy cows, *ACACA* expression initially decreased after short-term exposure (1H) but subsequently increased after longer exposure times (3H and 6H) (Table 8; Figure 8).

For *ACOX1* and *FASN*, gene upregulation was evident at 3H and became more pronounced at 6H, particularly at higher ZEN concentrations (Table 6 and Table 10; Figure 6 and Figure 10).

For *FADS2* expression, minor changes were observed with slight increases at intermediate doses and decreases at higher concentrations after 6H (Table 9; Figure 9). No consistent dose-dependent effect was observed for *ACAA1* and *HMGCR* (Table 7 and Table 11; Figure 7 and Figure 11).

The strongest suppression of *SC4MOL* was noted after 1H exposure, whereas the expression stabilized at 6H in both groups (Table 12, Figure 12).

The interaction between MS and ZEN affected the expression of *ACOX1*, *ACAA1*, *ACACA*, and *FASN* (Table 6, Table 7, Table 8 and Table 10). In healthy cows, *ACOX1* and *FASN* were more strongly upregulated, and *ACACA* showed a distinct early downregulation followed by recovery, while ketotic cows exhibited a more stable expression profile. No interaction was observed on *FADS2*, *HMGCR*, or *SC4MOL* (Table 9, Table 11 and Table 12).

## 3. Discussion

Ketosis in dairy cows is characterized by a negative energy balance, leading to increased mobilization of body fat and alterations in hepatic metabolism [17,18]. In our study, ketotic cows exhibited changes in the expression of genes related to glucose metabolism. Similar trends in gene expression have been reported previously, where ketosis was shown to reduce the expression of genes such as *ENO1*, *PGAM1*, *PGK1*, *TPI1*, and *PDHB*, all of which are integral to the glycolytic pathway [16].

These alterations suggest a shift towards anaerobic glycolysis and a potential impairment in the pyruvate dehydrogenase complex, which is crucial for linking glycolysis to the tricarboxylic acid (TCA) cycle [19]. As noted in previous transcriptomic studies, reduced *PDHB* expression may contribute to a bottleneck at the junction between glycolysis and the TCA cycle, limiting oxidative metabolism and ATP yield [16].

Regarding lipid metabolism, ketotic cows showed decreased expression of *ACACA*, a key enzyme in de novo fatty acid synthesis, and increased expression of *ACOX1*, involved in peroxisomal β-oxidation. This pattern is consistent with other reports showing suppressed lipogenesis and enhanced fatty acid oxidation in ketotic states [20]. In particular, increased *ACOX1* expression suggests a greater reliance on peroxisomal β-oxidation, which may serve to manage the influx of non-esterified fatty acids during energy deficiency [21].

ZEN is a mycotoxin known for its estrogenic effects, but it also influences hepatic metabolism and has a hepatotoxic effect [22]. Our findings demonstrate that ZEN exposure leads to dose-dependent changes in the expression of genes involved in both glucose and lipid metabolism. Other studies have also highlighted the impact of ZEN on hepatic gene expression, showing disruptions in pathways related to glucose absorption [23] and lipid synthesis [24], which may compromise liver metabolic homeostasis.

Notably, ZEN increased the expression of *PDHB* and *TPI1*, suggesting an upregulation of glycolytic flux. Conversely, it decreased the expression of *PGAM1* and *PGK1*, indicating a complex modulation of glycolysis. This dual regulation may reflect compensatory responses to metabolic disruption induced by ZEN exposure. Although comparable transcriptomic disruptions caused by ZEN have been reported in other species, such as zebrafish and pigs [22,25], for our knowledge, this is the first study to describe such effects in dairy cows.

In lipid metabolism, ZEN exposure resulted in increased expression of *ACOX1* and *FASN*, enzymes associated with fatty acid oxidation and synthesis, respectively. In the study by Jung et al. (2015) [26], cancer cells were shown to have elevated expression of *ACOX1* and *FASN*, particularly in brain metastases, suggesting a role in lipid metabolism alterations associated with disease progression. Interestingly, *ACACA* expression initially decreased after short-term exposure but increased with prolonged exposure, suggesting a biphasic response. Yang et al. (2017) [27], in a study on yaks, showed that higher dietary energy levels increased the expression of lipogenic genes, including *ACACA* and *FASN*, which may suggest similar regulatory mechanisms in response to ZEN. These findings align with previous studies indicating that ZEN can disrupt lipid metabolism and promote oxidative stress in hepatic cells. For instance, ZEN has been shown to induce oxidative stress in human hepatocytes, leading to DNA damage and modulation of stress-responsive genes [28].

The interaction between MS and ZEN exposure revealed that ketotic cows have a blunted transcriptional response compared to healthy cows. For instance, the upregulation of *PDHB* and *FASN* in response to ZEN was more pronounced in healthy cows, while ketotic cows exhibited a more stable expression profile. This may suggest that the metabolic flexibility of ketotic cows is compromised, limiting their ability to adapt to additional stressors, such as mycotoxin exposure. Supporting this, studies have indicated that cows with negative energy balance exhibit altered gene expression profiles [19], potentially affecting their response to toxins like ZEN.

Our findings suggest that the combined impact of ketosis and ZEN on gene expression, particularly for *PGAM1* and *ACACA*, is not merely additive but involves complex regulatory mechanisms. This interaction may increase the vulnerability of ketotic cows to the harmful effects of ZEN, posing a risk to liver function, productivity, and reproduction—especially considering the widespread occurrence of both ZEN contamination in feed [2] and ketosis [9] in high-producing dairy cows.

Our study also revealed that ZEN exposure duration significantly influenced gene expression, with the strongest effects—including MS × ZEN interactions—appearing after just 1 h. Key metabolic genes (e.g., *FASN*, *PDHB*, *PGAM1*, *ACAA1*, *PGK1*) showed marked changes, especially in healthy cows, indicating rapid disruption of lipid and glucose metabolism (such as fatty acid synthesis and oxidation, glycolysis and pyruvate oxidation). These effects may suggest that the transcriptional response to ZEN is modulated by MS, reflecting differences in energy availability and regulatory capacity

Although we did not directly calculate genetic or phenotypic correlations in this in vitro model, the expression patterns observed are consistent with findings from previous in vivo studies. For example, Loor et al. (2007) [16] reported altered hepatic expression of metabolic genes, including *FASN* and *PDHB*, in cows with nutrition-induced ketosis, which supports our observation of MS-dependent expression shifts. Similarly, Taniguchi et al. (2008) [29] demonstrated that gene expression profiles in adipose tissue vary with physiological and metabolic status, reinforcing the idea that individual metabolic background influences transcriptional responses. Thus, our results align with the literature and suggest that even short-term ZEN exposure can interact with underlying metabolic traits to rapidly alter gene regulation.

With prolonged exposure (3 or 6H), differences between groups began to diminish. Many genes showed stabilization or normalization of expression (e.g., *ACAA1*, *ENO1*, *SC4MOL*), suggesting a potential adaptive or homeostatic response to the toxin. In some cases (e.g., *ACACA*, *TPI1*), healthy hepatocytes exhibited compensatory upregulation, possibly reflecting cellular attempts to restore metabolic balance. Conversely, ketotic cows maintained a more muted transcriptional profile, particularly for *PGK1* and *PGAM1*, suggesting a limited adaptive capacity.

It is important to note that this research was conducted in vitro and thus lacks the complexity of whole-organism responses, including hormonal, immune, and microbiome-mediated interactions. The advantages and limitations of in vitro approaches in toxicological studies have been widely discussed by researchers, as comprehensively summarized by Eisenbrand et al. (2002) [30] in their review. Moreover, the sample size was limited, as liver cells were derived from a relatively small number of cows, which reflects the technical and ethical constraints associated with primary hepatocyte isolation from clinically defined dairy cows. Despite this, clear and consistent transcriptional patterns were observed, supporting the robustness of the conclusions. Additionally, as feed was not tested for zearalenone, background exposure cannot be fully excluded; however, all cows were kept under identical conditions, and untreated hepatocytes served as internal controls. These are acknowledged limitations; however, the observed transcriptional effects are robust and consistent, strongly supporting the experimental hypothesis. Future studies should also consider the combined effects of multiple mycotoxins, which more closely reflect real-life exposure scenarios but were beyond the scope of this focused experimental design.

Moreover, this study was designed to assess the direct transcriptional responses of hepatocytes to zearalenone without the influence of metabolic interventions. While this approach allowed for clearer interpretation of zearalenone-specific effects, co-treatment with metabolic agents, such as propylene glycol or other glucogenic compounds, may alter cellular responsiveness, particularly in ketotic conditions. Exploring such interactions could provide valuable insights for practical mycotoxin mitigation strategies in dairy cows and should be considered in future studies.

Finally, this study presents a novel in vitro model for evaluating the metabolic effects of mycotoxins in cows with differing energy status. To our knowledge, this is the first application of this model. The observed gene-specific changes in expression patterns—strongly dependent on both ZEN dose and MS—confirm the model’s biological responsiveness and utility. These results not only validate the experimental approach but also provide a valuable foundation for future in vivo studies exploring the interplay between metabolic disorders and environmental toxins in dairy production.

## 4. Materials and Methods

### 4.1. Animals

This study was conducted between 2018 and 2020, following ethical approval granted in 2017 (permission number: 124/2017). The protocol did not undergo any modifications, and all procedures were performed within the validity period of the original approval. The study involved a total of 12 early lactation dairy cows. The animals were aged between 4 and 6 years and had an average body weight of approximately 725 kg. On the 10th day in milk (DIM), the cows were categorized into two groups based on blood β-hydroxybutyrate (BHB) concentrations: healthy cows, characterized by BHB less than 1.2 mmol/L, and cows in clinical ketosis, with BHB greater than 3.0 mmol/L, based on the thresholds proposed by Oetzel (2004) [9]. Assessment of BHB was conducted using blood samples collected from the tail vein 4 to 6 h after morning feeding, and BHB was determined with the Optimum Xido (Abbott Diabetes Care, Abbott Park, IL, USA) glucometer [31]. Cows were fed a standard fresh total mixed ration (TMR) diet (17% CP, 30% NDF, 30% starch in DM), based on maize silage (40% of diet DM) and including ensiled high-moisture corn grain (20% of diet DM). Neither the diet nor its components were tested for mycotoxin contamination.

For gene expression analysis related to glucose metabolism (*ENO1*, *PDHB*, *PGAM1*, *PGK1*, *TPI1*) three healthy cows (mean BHB = 0.7 mmol/L) and three ketotic cows (mean BHB = 4.33 mmol/L) were used. For gene expression analysis related to lipid metabolism (*ACOX1*, *ACAA1*, *ACACA*, *FADS2*, *FASN*, *HMGCR*, *SC4MOL*), a different set of three healthy cows (mean BHB = 0.76 mmol/L) and three ketotic cows (mean BHB = 3.76 mmol/L) were selected. Thus, in total, samples from 12 animals were used in the study.

### 4.2. Liver Biopsies

Liver tissue samples (from 0.5 to 1.5 g) were obtained on 10 DIM through liver biopsies performed by a veterinary surgeon following the method described by Van den Top et al. (1998) [32]. Reusable biopsy instruments were used, including a cannula (0.9 cm diameter) with a trocar-tipped length of 52 cm and a 0.8 cm diameter stainless steel stylet. The biopsy was conducted at the 11th intercostal space along the line between the elbow and hook. A skin area of 5 × 5 cm was clipped and disinfected with 70% ethanol and a 3% iodine solution. Local anesthesia was administered using 10 mL of 2% Polocainum hydrochloricum cum adrenalino (Biowet, Puławy, Poland). After a stab incision, the biopsy needle was inserted towards the opposite elbow to access the liver. Biopsies were performed under ultrasound guidance to obtain samples from the caudate lobe.

### 4.3. Isolation of Hepatocytes

Hepatocyte isolation was performed using a combination of the simplified manual perfusion method described by Panda et al. (2015) [33] and the non-perfusion technique for cell isolation described by Spotorno et al. (2006) [34], with slight modifications. Liver tissue obtained through biopsy was aseptically transferred to Dulbecco’s phosphate buffered saline (DPBS, Gibco, Billings, MT, USA) containing an antibiotic–antimycotic solution (Sigma Aldrich, St. Louis, MO, USA) and transported on ice to the laboratory within 60 min.

In a sterile environment, liver samples were manually injected with 100 mL of pre-cooled (4 °C) Ca^2+^ and Mg^2+^ free 33 mM 4-(2-hydroxyethyl)-1-piperazineethanesulfonic acid (HEPES buffer, pH 7.6, Gibco) containing 0.5 mM Ethylene glycol-bis(β-aminoethyl ether)-N,N,N′,N′-tetraacetic acid (EGTA, Sigma Aldrich) to remove blood clots. The tissue was minced, homogenized, and washed several times with DPBS without EGTA. After a final wash, the samples were transferred to a flask containing a collagenase solution (Collagenase II, 100 U/mL in HBSS-HEPES buffer) and gently stirred for 12 min. Fetal bovine serum (FBS, Cytogen, Seoul, Republic of Korea) was then added to the cell suspension along with ice-cold HBSS, followed by filtration through cheesecloth. The cell suspension was centrifuged at 150× *g* for 5 min at 4 °C with DNase solution. The pellet was washed with DPBS and centrifuged twice more. The final cell pellet was resuspended in DPBS.

### 4.4. Experimental Design and Cell Culture

For each gene analysis, cell cultures were established using liver biopsies from healthy or ketotic cows (n = 3 per group). Cells were seeded on dry collagen-coated plates at a density of 4.5 × 10^4^ viable cells/cm^2^ in a growth medium supplemented with 10% FBS. The process of hepatocyte isolation and cell culture used in this study was previously developed and optimized by our team and published in Barć et al. (2023) [15]. Cultures were maintained at 38.5 °C in a humidified atmosphere with 5% CO_2_ for 24 h to allow cell attachment. After 24 h, the medium was replaced, marking the start of the exposure period. Cells were exposed to ZEN (Sigma Aldrich) at concentrations of 0, 10, 20, 50, and 100 µM for 1, 3, and 6 h. The selected exposure times were based on our previous work, which demonstrated that bovine hepatocytes remain viable and metabolically active for a limited period under standard in vitro conditions [15]. Reliable transcriptomic responses can be captured within the first few hours of exposure, while viability and functional activity tend to decline beyond 6 h. The chosen concentration range (10–100 µM) was based on the literature data from similar in vitro toxicological studies [35,36] and was confirmed in preliminary viability assays to induce transcriptional changes [15]. Each dose and time combination was performed in quadruplicate to increase reproducibility.

### 4.5. RNA Isolation and qPCR Analysis

RNA isolation and cDNA synthesis were performed using the TaqMan Gene Expression Cells-to-CT kit (Applied Biosystems, Foster City, CA, USA) according to the manufacturer’s protocol. The resulting pre-amplified cDNA preparations were analyzed by real-time PCR in a StepOnePlus Real-time PCR System (Applied Biosystems) using TaqMan Gene Expression Assays and TaqMan Gene Expression Master Mix containing ROX (Applied Biosystems, Foster City, CA, USA) for the following genes: *ENO1* (Bt03230937_m1), *PDHB* (Bt03269405_m1), *PGAM1* (Bt03225424_g1), *PGK1* (Bt03225857_m1), *TPI1* (Bt03224990_m1), *ACOX1* (Bt03244689_m1), *ACAA1* (Bt03221026_m1), *ACACA* (Bt03213371_m1), *FADS2* (Bt03256252_m1), *FASN* (Bt03210481_m1), *HMGCR* (Bt03258816_m1) and *SC4MOL* (Bt03258188_m1). The following PCR conditions were used: incubation for 2 min at 50 °C followed by incubation for 10 min at 95 °C and 40 cycles (denaturation step: 15 s at 95 °C; annealing/elongation step: 60 s at 60 °C). Obtained results were normalized to the *GAPDH* (Bt03210912_g1) as a reference gene.

### 4.6. Statistical Analysis

Prior to statistical analysis, the distribution of all variables was examined. Given the relatively small number of observations, the Shapiro–Wilk test was used to assess normality, employing the PROC UNIVARIATE procedure with the NORMAL option (SAS v.9.4). The test results indicated no significant deviations from normality for any of the variables (*p* > 0.05). Extreme values and quartiles were calculated using PROC UNIVARIATE, and, based on these, the presence of outliers was assessed using the 1.5× IQR criterion. No outliers were detected in the dataset. For each variable, analysis of variance (ANOVA) was performed using a fixed linear model that included metabolic status (MS; healthy/ketotic), ZEN dose (ZEN), the interaction between MS and ZEN, and the cow effect nested within MS. The effect of time was not included in the model. Measurements taken at different time points (1 h, 3 h, and 6 h) were treated as separate variables and analyzed using the same linear model. The PROC GLM procedure was used for ANOVA computations. Least squares means were calculated for the effects of MS, ZEN, and their interaction. The significance of differences between factor levels was evaluated using the PDIFF option with the Tukey–Kramer correction for multiple comparisons.

## 5. Conclusions

Our study highlights the intricate interplay between metabolic status and Zearalenone exposure in dairy cows. Ketosis alters the hepatic gene expression profile, reducing the liver’s capacity to respond to additional challenges such as ZEN. A study has proved that ZEN poses a health risk to dairy cows, particularly those experiencing metabolic disorders such as ketosis. This underscores the importance of integrated management practices that address both nutritional and environmental factors to safeguard dairy cow health and productivity.

## Figures and Tables

**Table 1:** Relative mRNA expression of *ENO1* in hepatocyte cultures isolated from healthy and ketotic cows exposed to various concentrations of Zearalenone (ZEN; 10, 20, 50, 100 μM) for 1, 3, and 6 h. Gene expression was normalized to the control group (0 μM) using the 2^(−ΔΔCT)^ method. Values represent least squares mean (LSM), and SEM denotes the standard error of the mean. Statistical significance was determined for the effects of metabolic status (MS), ZEN (Z), and their interaction (MS*Z).

*ENO1*	Healthy Cows					Ketotic Cows					SEM	*p* Value		
ZEN Dose [μM]	0	10	20	50	100	0	10	20	50	100		MS	Z	MS*Z
1H	1.04	0.97	0.95	1.16	1.15	0.99	1.04	0.98	1.03	1.06	0.04	0.2073	0.0114	0.1323
3H	1.03	1.15	1.12	1.04	1.16	1.02	0.97	0.98	1.03	1.03	0.04	0.002	0.4216	0.1566
6H	1.03	0.96	1.11	1.04	1.05	1.02	1.00	1.00	1.06	1.02	0.03	0.4741	0.2163	0.2433

**Table 2:** Relative mRNA expression of *PDHB* in hepatocyte cultures isolated from healthy and ketotic cows exposed to various concentrations of Zearalenone (ZEN; 10, 20, 50, 100 μM) for 1, 3, and 6 h. Gene expression was normalized to the control group (0 μM) using the 2^(−ΔΔCT)^ method. Values represent least squares mean (LSM), and SEM denotes the standard error of the mean. Statistical significance was determined for the effects of metabolic status (MS), ZEN (Z), and their interaction (MS*Z). Different letters within the same row indicate statistically significant differences between ZEN doses at the same incubation time (*p* < 0.05).

*PDHB*	Healthy Cows					Ketotic Cows					SEM	*p* Value		
ZEN Dose [μM]	0	10	20	50	100	0	10	20	50	100		MS	Z	MS*Z
1H	1.01 ^a^	1.13 ^a^	1.45 ^b^	1.57 ^b^	1.68 ^b^	1.00 ^a^	1.04 ^a^	1.02 ^a^	1.02 ^a^	1.01 ^a^	0.09	<0.0001	0.0077	0.0070
3H	0.97	1.10	1.52	1.40	1.72	1.00	0.98	1.41	1.52	1.47	0.08	0.2594	<0.0001	0.2704
6H	1.01	1.24	1.60	1.53	1.62	0.99	1.11	1.41	1.40	1.38	0.09	0.0239	<0.0001	0.8112

**Table 3:** Relative mRNA expression of *PGAM1* in hepatocyte cultures isolated from healthy and ketotic cows exposed to various concentrations of Zearalenone (ZEN; 10, 20, 50, 100 μM) for 1, 3, and 6 h. Gene expression was normalized to the control group (0 μM) using the 2^(−ΔΔCT)^ method. Values represent least squares mean (LSM), and SEM denotes the standard error of the mean. Statistical significance was determined for the effects of metabolic status (MS), ZEN (Z), and their interaction (MS*Z). Different letters within the same row indicate statistically significant differences between ZEN doses at the same incubation time (*p* < 0.05).

*PGAM1*	Healthy Cows					Ketotic Cows					SEM	*p* Value		
ZEN Dose [μM]	0	10	20	50	100	0	10	20	50	100		MS	Z	MS*Z
1H	1.00 ^a^	0.99 ^a^	0.80 ^bcd^	0.76 ^bc^	0.71 ^b^	1.01 ^a^	1.02 ^a^	1.02 ^a^	1.02 ^a^	0.98 ^acd^	0.05	0.0001	0.0169	0.0393
3H	1.02	0.91	0.76	0.79	0.72	1.02	1.00	0.89	0.64	0.60	0.06	0.757	<0.0001	0.0774
6H	1.00 ^ab^	1.02 ^a^	0.83 ^bc^	0.68 ^c^	0.75 ^c^	1.01 ^ab^	0.98 ^ab^	0.9 ^ab9^	1.05 ^a^	0.87 ^abc^	0.04	0.0003	0.0011	0.0020

**Table 4:** Relative mRNA expression of *PGK1* in hepatocyte cultures isolated from healthy and ketotic cows exposed to various concentrations of Zearalenone (ZEN; 10, 20, 50, 100 μM) for 1, 3, and 6 h. Gene expression was normalized to the control group (0 μM) using the 2^(−ΔΔCT)^ method. Values represent least squares mean (LSM), and SEM denotes the standard error of the mean. Statistical significance was determined for the effects of metabolic status (MS), ZEN (Z), and their interaction (MS*Z). Different letters within the same row indicate statistically significant differences between ZEN doses at the same incubation time (*p* < 0.05).

*PGK1*	Healthy Cows					Ketotic Cows					SEM	*p* Value		
ZEN Dose [μM]	0	10	20	50	100	0	10	20	50	100		MS	Z	MS*Z
1H	1.04 ^ab^	0.96 ^a^	0.98 ^ab^	1.21 ^b^	1.22 ^b^	1.01 ^ab^	1.02 ^a^	0.99 ^ab^	1.00 ^ab^	1.01 ^ab^	0.03	0.0028	0.0026	0.0021
3H	1.01	0.85	0.83	0.71	0.72	1.03	0.83	0.82	0.82	0.76	0.05	0.4089	0.0005	0.7145
6H	0.99 ^ab^	0.95 ^ab^	0.95 ^ab^	0.72 ^cd^	0.53 ^e^	1.00 ^ab^	1.04 ^b^	0.84 ^ad^	0.85 ^acd^	0.68 ^ce^	0.04	0.0425	<0.0001	0.0366

**Table 5:** Relative mRNA expression of *TPI1* in hepatocyte cultures isolated from healthy and ketotic cows exposed to various concentrations of Zearalenone (ZEN; 10, 20, 50, 100 μM) for 1, 3, and 6 h. Gene expression was normalized to the control group (0 μM) using the 2^(−ΔΔCT)^ method. Values represent low least squares mean (LSM), and SEM denotes the standard error of the mean. Statistical significance was determined for the effects of metabolic status (MS), ZEN (Z), and their interaction (MS*Z).

*TPI1*	Healthy Cows					Ketotic Cows					SEM	*p* Value		
ZEN Dose [μM]	0	10	20	50	100	0	10	20	50	100		MS	Z	MS*Z
1H	0.98	0.91	1.07	1.21	1.21	1.00	1.03	0.99	1.23	1.14	0.07	0.9959	0.0122	0.6842
3H	1.01	0.96	1.10	0.95	1.07	1.02	1.03	0.98	0.96	1.02	0.04	0.5375	0.2019	0.1878
6H	1.01	0.97	1.20	1.14	1.35	1.01	1.00	1.22	1.26	1.29	0.06	0.5881	0.0003	0.7214

**Table 6:** Relative mRNA expression of *ACOX1* in hepatocyte cultures isolated from healthy and ketotic cows exposed to various concentrations of zearalenone (ZEN; 10, 20, 50, 100 μM) for 1, 3, and 6 h. Gene expression was normalized to the control group (0 μM) using the 2^(−ΔΔCT)^ method. Values represent means, and SEM denotes the standard error of the mean. Statistical significance was determined for the effects of metabolic status (MS), ZEN dose (Z), and their interaction (MS*Z). Different letters within the same row indicate statistically significant differences between ZEN doses at the same incubation time (*p* < 0.05).

*ACOX1*	Healthy Cows					Ketotic Cows					SEM	*p* Value		
ZEN Dose [μM]	0	10	20	50	100	0	10	20	50	100		MS	Z	MS*Z
1H	1.06 ^a^	1.03 ^a^	1.19 ^a^	1.36 ^a^	2.30 ^b^	1.02 ^a^	1.06 ^a^	0.91 ^a^	1.03 ^a^	1.03 ^a^	0.16	0.0016	0.0042	0.0049
3H	1.01 ^a^	1.27 ^bc^	1.43 ^cd^	1.40 ^bcd^	1.58 ^d^	1.00 ^a^	1.02 ^a^	1.18 ^ab^	1.35 ^bcd^	1.46 ^cd^	0.06	0.0029	<0.0001	0.2485
6H	1.07 ^a^	1.11 ^a^	1.47 ^b^	1.43 ^b^	1.61 ^b^	1.01 ^a^	1.01 ^a^	1.01 ^a^	1.36 ^b^	1.43 ^b^	0.07	0.0012	<0.0001	0.0658

**Table 7:** Relative mRNA expression of *ACAA1* in hepatocyte cultures isolated from healthy and ketotic cows exposed to various concentrations of zearalenone (ZEN; 10, 20, 50, 100 μM) for 1, 3, and 6 h. Gene expression was normalized to the control group (0 μM) using the 2^(−ΔΔCT)^ method. Values represent means, and SEM denotes the standard error of the mean. Statistical significance was determined for the effects of metabolic status (MS), ZEN dose (Z), and their interaction (MS*Z). Different letters within the same row indicate statistically significant differences between ZEN doses at the same incubation time (*p* < 0.05).

*ACAA1*	Healthy Cows					Ketotic Cows					SEM	*p* Value		
ZEA Dose [μM]	0	10	20	50	100	0	10	20	50	100		MS	Z	MS*Z
1H	1.01 ^a^	1.00 ^a^	1.01 ^a^	1.00 ^a^	0.75 ^b^	1.01 ^a^	1.02 ^a^	0.98 ^a^	1.01 ^a^	1.05 ^a^	0.02	0.0008	0.0004	<0.0001
3H	1.01	1.05	1.00	1.02	1.04	0.99	1.02	1.01	1.05	1.02	0.03	0.8667	0.6951	0.8013
6H	0.99	0.95	1.01	1.01	1.02	1.00	0.95	1.01	0.99	1.02	0.02	0.8639	0.0444	0.9812

**Table 8:** Relative mRNA expression of *ACACA* in hepatocyte cultures isolated from healthy and ketotic cows exposed to various concentrations of zearalenone (ZEN; 10, 20, 50, 100 μM) for 1, 3, and 6 h. Gene expression was normalized to the control group (0 μM) using the 2^(−ΔΔCT)^ method. Values represent means, and SEM denotes the standard error of the mean. Statistical significance was determined for the effects of health status (MS), ZEN dose (Z), and their interaction (MS*Z). Different letters within the same row indicate statistically significant differences between ZEN doses at the same incubation time (*p* < 0.05).

*ACACA*	Healthy Cows					Ketotic Cows					SEM	*p* Value		
ZEN Dose [μM]	0	10	20	50	100	0	10	20	50	100		MS	Z	MS*Z
1H	1.01 ^ab^	0.83 ^acde^	0.81 ^ce^	0.82 ^cde^	0.74 ^e^	1.01 ^abd^	1.00 ^abcd^	1.02 ^ab^	0.99 ^abcd^	1.04 ^b^	0.04	<0.0001	0.0301	0.0119
3H	1.04 ^acd^	0.98 ^abc^	0.90 ^ab^	1.23 ^cd^	1.24 ^d^	1.01 ^abcd^	0.93 ^ab^	0.75 ^b^	0.74 ^b^	0.77 ^b^	0.03	<0.0001	0.0002	<0.0001
6H	1.02	0.96	1.09	1.15	1.20	1.02	1.01	1.01	0.96	1.01	0.03	0.0013	0.0393	0.009

**Table 9:** Relative mRNA expression of *FADS2* in hepatocyte cultures isolated from healthy and ketotic cows exposed to various concentrations of zearalenone (ZEN; 10, 20, 50, 100 μM) for 1, 3, and 6 h. Gene expression was normalized to the control group (0 μM) using the 2^(−ΔΔCT)^ method. Values represent means, and SEM denotes the standard error of the mean. Statistical significance was determined for the effects of metabolic status (MS), ZEN dose (Z), and their interaction (MS*Z). Different letters within the same row indicate statistically significant differences between ZEN doses at the same incubation time (*p* < 0.05).

*FADS2*	Healthy Cows					Ketotic Cows					SEM	*p* Value		
ZEN Dose [μM]	0	10	20	50	100	0	10	20	50	100		MS	Z	MS*Z
1H	0.99	1.01	1.01	1.05	0.99	0.99	1.01	1.05	1.05	0.98	0.03	0.8029	0.1622	0.8925
3H	1.02	1.01	1.01	1.03	1.03	1.00	0.98	1.04	1.00	1.04	0.03	0.582	0.5647	0.7711
6H	1.02 ^a^	1.04 ^a^	0.99 ^a^	0.77 ^b^	0.73 ^b^	1.01 ^a^	0.98 ^a^	1.05 ^a^	0.81 ^a^	0.66 ^b^	0.04	0.7861	<0.0001	0.5092

**Table 10:** Relative mRNA expression of *FASN* in hepatocyte cultures isolated from healthy and ketotic cows exposed to various concentrations of zearalenone (ZEN; 10, 20, 50, 100 μM) for 1, 3, and 6 h. Gene expression was normalized to the control group (0 μM) using the 2^(−ΔΔCT)^ method. Values represent means, and SEM denotes the standard error of the mean. Statistical significance was determined for the effects of metabolic status (MS), ZEN dose (Z), and their interaction (MS*Z). Different letters within the same row indicate statistically significant differences between ZEN doses at the same incubation time (*p* < 0.05).

*FASN*	Healthy Cows					Ketotic Cows					SEM	*p* Value		
ZEN Dose [μM]	0	10	20	50	100	0	10	20	50	100		MS	Z	MS*Z
1H	1.06	0.75	0.99	0.92	0.99	0.99	1.02	1.03	1.00	0.99	0.04	0.0226	0.0295	0.0122
3H	1.03 ^a^	1.01 ^a^	1.24 ^a^	1.28 ^a^	1.62 ^b^	1.01 ^a^	1.06 ^a^	0.99 ^a^	1.06 ^a^	1.03 ^a^	0.06	0.0001	0.0014	0.0015
6H	1.02	0.99	1.46	1.70	1.77	0.99	1.03	1.56	1.67	1.55	0.07	0.5961	<0.0001	0.2552

**Table 11:** Relative mRNA expression of *HMGCR* in hepatocyte cultures isolated from healthy and ketotic cows exposed to various concentrations of zearalenone (ZEN; 10, 20, 50, 100 μM) for 1, 3, and 6 h. Gene expression was normalized to the control group (0 μM) using the 2^(−ΔΔCT)^ method. Values represent means, and SEM denotes the standard error of the mean. Statistical significance was determined for the effects of metabolic status (MS), ZEN dose (Z), and their interaction (MS*Z). Different letters within the same row indicate statistically significant differences between ZEN doses at the same incubation time (*p* < 0.05).

*HMGCR*	Healthy Cows					Ketotic Cows					SEM	*p* Value		
ZEN Dose [μM]	0	10	20	50	100	0	10	20	50	100		MS	Z	MS*Z
1H	1.03 ^a^	0.83 ^ab^	0.80 ^ab^	0.82 ^ab^	0.76 ^ab^	1.00 ^a^	1.00 ^a^	0.86 ^ab^	0.81 ^ab^	0.70 ^b^	0.06	0.4948	0.0023	0.3555
3H	1.01 ^a^	1.04 ^a^	0.86 ^ab^	1.05 ^a^	0.95 ^ab^	1.01 ^a^	1.02 ^a^	0.94 ^ab^	0.99 ^a^	0.87 ^ab^	0.05	0.6241	0.0343	0.4995
6H	0.99	0.82	1.12	0.94	1.07	1.01	1.01	1.01	0.98	0.99	0.09	0.8445	0.5107	0.4959

**Table 12:** Relative mRNA expression of *SC4MOL* in hepatocyte cultures isolated from healthy and ketotic cows exposed to various concentrations of zearalenone (ZEN; 10, 20, 50, 100 μM) for 1, 3, and 6 h. Gene expression was normalized to the control group (0 μM) using the 2^(−ΔΔCT)^ method. Values represent means, and SEM denotes the standard error of the mean. Statistical significance was determined for the effects of health metabolic status (MS), ZEN dose (Z), and their interaction (MS*Z). Different letters within the same row indicate statistically significant differences between ZEN doses at the same incubation time (*p* < 0.05).

*SC4MOL*	Healthy Cows					Ketotic Cows					SEM	*p* Value		
ZEN Dose [μM]	0	10	20	50	100	0	10	20	50	100		MS	Z	MS*Z
1H	1.00 ^a^	1.03 ^a^	0.69 ^bc^	0.73 ^b^	0.64 ^c^	1.00 ^a^	0.96 ^a^	0.78 ^ab^	0.61 ^c^	0.60 ^c^	0.06	0.4321	<0.0001	0.3962
3H	1.00 ^a^	0.79 ^ab^	0.72 ^ab^	0.72 ^ab^	0.70 ^ab^	1.00 ^a^	0.78 ^ab^	0.71 ^ab^	0.71 ^ab^	0.70 ^ab^	0.06	0.9359	0.0013	0.9998
6H	1.00	0.98	1.01	1.01	0.97	1.01	1.02	1.00	0.98	1.02	0.03	0.611	0.9917	0.6676

## Data Availability

Data available upon request.

## Abbreviations

The following abbreviations are used in this manuscript:

*ACAA1*	Acetyl-CoA Acyltransferase 1
*ACACA*	Acetyl-CoA Carboxylase Alpha
*ACOX1*	Acyl-CoA Oxidase 1
ANOVA	Analysis of variance
BHB	β-hydroxybutyrate
DIM	Day in milk
DPBS	Dulbecco’s Phosphate-Buffered Saline
EGTA	Ethylene glycol-bis(β-aminoethyl ether)-N,N,N′,N′-tetraacetic acid
*ENO1*	Enolase 1
*FADS2*	Fatty Acid Desaturase 2
*FASN*	Fatty Acid Synthase
FBS	Fetal Bovine Serum
HEPES	4-(2-hydroxyethyl)-1-piperazineethanesulfonic acid
HBSS	Hank’s Balanced Salt Solution
*HMGCR*	3-Hydroxy-3-Methylglutaryl-CoA Reductase
MS	Metabolic status
*PDHB*	Pyruvate Dehydrogenase E1 Subunit Beta
PDIFF	Probability of difference
*PGAM1*	Phosphoglycerate Mutase 1
*PGK1*	Phosphoglycerate Kinase 1
*SC4MOL*	Sterol-C4-Methyl Oxidase-Like
*TPI1*	Triosephosphate Isomerase 1
ZEN	Zearalenone
